# Contrast‐enhanced ultrasound is useful for the evaluation of focal liver lesions in children

**DOI:** 10.1002/ajum.12279

**Published:** 2021-06-27

**Authors:** Alvaro Torres, Seppo K. Koskinen, Henrik Gjertsen, Björn Fischler

**Affiliations:** ^1^ Department of Clinical Science, Intervention and Technology (CLINTEC) Karolinska Institute Alfred Nobels alle 8 Huddinge 141 52 Sweden; ^2^ Division of Radiology Karolinska University Hospital C1:46 Huddinge Sweden; ^3^ Division of Transplantation Surgery Karolinska University Hospital Huddinge Stockholm 141 86 Sweden; ^4^ Division of Paediatrics Karolinska University Hospital, Novum Blickagången 6a Huddinge 141 57 Sweden

**Keywords:** contrast media, gastroenterology, off‐label use, paediatrics, ultrasonography

## Abstract

**Introduction:**

Contrast‐enhanced ultrasound (CEUS) is a widely used diagnostic method. In adults, it has been proven to be a useful alternative to CT and MRI for the characterisation of focal liver lesions (FLLs). However, since there is no official paediatric licensing for any ultrasound contrast agents in Europe, its use has been restricted.

**Purpose:**

To retrospectively outline our experience with CEUS as a tool for the characterisation of FLLs in paediatric patients.

**Methods:**

An eleven‐year retrospective single‐centre study. During this period, we identified 287 CEUS examinations performed on children, of these 36 were relevant first‐time examinations with the aim of characterising a focal liver lesion. Clinical and radiological data were collected from the hospital chart.

**Results:**

The overall agreement between the CEUS diagnosis and the reference diagnosis for benign versus malignant differentiation was 75%. When analysing conclusive CEUS examinations only, the overall agreement was 96%. The specificity for correctly characterising a lesion as benign was 96%, and the negative predictive value was 100%. No side effects from CEUS were detected.

**Conclusions:**

Our study reinforces that CEUS can be useful in the medical workup for the identification and classification of focal liver lesions in children.

## Introduction

Liver tumours are rare in children, but when suspected they can pose a diagnostic challenge. Several factors, such as the patient's age and whether or not the liver is cirrhotic, are to be taken into account when considering differential diagnostics.^1^ Imaging is one of the cornerstones of the workup for correct diagnosis in these patients. Available methods include contrast‐enhanced computer tomography (CECT) and contrast‐enhanced magnetic resonance imaging (CEMRI). These methods provide a great deal of information but are also associated with risks and disadvantages. In CECT, there is a considerable degree of harmful radiation^2^, ^3^ and also potentially nephrotoxic iodinated contrast media is administered.^4^ MRI usually requires sedation or general anaesthesia due to long examination times since the child needs to lie still for the complete duration of the examination. Ultrasonography (US), on the contrary, is radiation‐free, does not require the patient to be completely still and is portable and fast. Therefore, it is one of the most commonly used imaging methods in paediatric radiology. However, it is often not sensitive enough to characterise a focal liver lesion (FLL). In order to obtain more diagnostic information, intravenous injection of hexafluoride microbubbles (SonoVue®, Bracco, Milan) while scanning, that is a contrast‐enhanced ultrasound (CEUS), may be performed. This contrast agent is not nephrotoxic, as it is eliminated by the lungs with the expired air. CEUS examinations almost never require sedation.^5^ CEUS can be used as a complement or sometimes as an alternative to CT or MRI for FLL characterisation.^6^ So far, scientific reports and clinical guidelines support its use for this purpose, in both adults and children.^5^, ^6^, ^7^

Exclusively paediatric original studies in this field are very few. Most studies have been performed on adults, and the incidence and type of tumours in children differ significantly from those in adults.^1^, ^8^ According to the European Federation of Societies for Ultrasound in Medicine and Biology (EFSUMB) position statement on paediatric CEUS from 2017,^5^ only four studies on 125 children in total deal with this subject directly. A literature search on PubMed for studies published after 2017 yielded one additional study which included 31 cases of focal liver lesions in children.^9^

Recently in the United States, an ultrasound contrast agent by the name of Lumason® (Sulfur Hexafluoride), a rebranding of SonoVue® (Sulfur Hexafluoride), received FDA approval for hepatic use in children.^10^ Nevertheless, in Europe and many other parts of the world, the use of CEUS for any paediatric application is still considered off‐label, due to the absence of official licensing.^11^ This is most likely a restrictive factor for its use. Therefore, every report regarding paediatric use of CEUS is of importance.

The purpose of our study was to retrospectively outline our experience with CEUS as a tool for the characterisation of FLLs in paediatric patients.

## Methods and Materials

**Figure 1:**
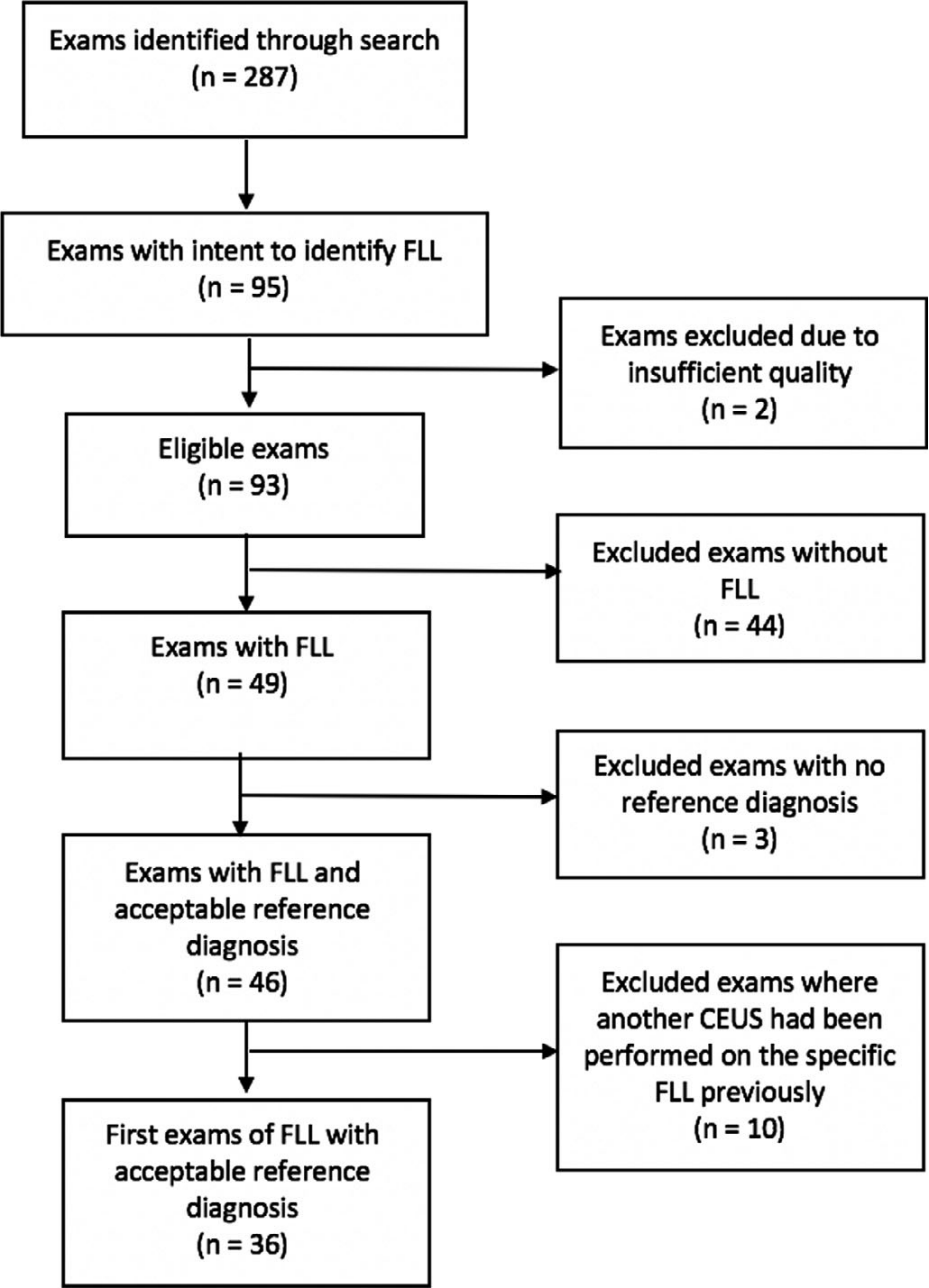
Flow chart of the selection process. FLL, Focal Liver Lesion.

All cases were gathered from *Karolinska University Hospital, Huddinge*, which serves as a tertiary referral centre for paediatric and adult hepatology. Through the hospital PACS (picture archiving and communications system), we identified a total of 10681 ultrasound examinations performed on children (<18 years old) between 1 January 2004 and 31 December 2014, of which 287 were CEUS examinations. Out of these, we identified 95 CEUS examinations with the intent to identify the presence of an FLL or to characterise a previously visualised FLL. Figure 1 illustrates the selection process for the examinations included in our study. Out of the initial 95 examinations, two were excluded because they did not yield images of sufficient diagnostic quality for the radiologist to be able to produce a report. Another 44 examinations in fact showed no FLL and were not included. Furthermore, three of the remaining 49 examinations did not have any acceptable reference diagnosis and were excluded. Five of the patients had one or more CEUS examinations performed as follow‐up examinations on one and the same FLL, previously characterised by CEUS. We only included the first diagnostic examination of each patient in our study and therefore 10 follow‐up examinations where excluded. In one case, two CEUS examinations on one single patient were included, since the focus of the CEUS was two different FLLs and the two examinations were performed five years apart. We therefore chose to consider these as two first‐time examinations.

This left us with 36 first‐time examinations with intent to characterise a FLL, performed on 35 patients.

For the CEUS diagnosis, the original radiology report was used. In cases where an addition to, or correction of the report was made, the last version of the report was used. As the method is largely operator dependent, and no standardised archiving of contrast‐enhanced images or cineloops were in use, no general secondary evaluation of the stored images and cineloops was performed to assess interobserver reliability. However, one specific case required closer attention and clarification. This case was re‐evaluated by the senior CEUS radiologist in our institution. He was instructed to review the case by looking through the stored images and cineloops and was instructed to use all available clinical information and previous imaging performed on the patient up until the date of the original CEUS, at his own discretion. He was then asked to produce a report of his findings.

Accessing the hospital electronic chart system, clinical and anthropometric data were retrospectively collected. Additionally, information on CEUS examination, biopsy as well as MRI and/or CT results was collected and used for determining the reference for comparison.

Reference standard was histology. If no histology was available, follow‐up of at least two years with radiological imaging, clinical follow‐up without radiologic imaging of at least two years or follow‐up with radiologic imaging of less than two years was used, in falling order of priority.

The results were stratified into two groups: one with cirrhosis at the time of the CEUS investigation and one without cirrhosis. Cirrhosis was defined as a histopathologically confirmed diagnosis.

We calculated agreement between CEUS diagnosis and reference diagnosis as well as specificity and negative predictive value (NPV) when applicable.

During the period of the study, the local standard for dose calculation was 0.1 ml/kg up to 24 kg where the full dose of 2.4 ml was administered.

The regional ethical review board approved this study.

### Ethical Approval

This study has been reviewed by the local ethics committee and has therefore been performed in accordance with the ethical standards laid down in the Declaration of Helsinki (as revised in Brazil 2013).

## Results

Descriptive data for the examinations are presented in Table 1.

**Table 1:** Anthropometric data

Parameter	Data (Mean (range)) unless otherwise stated
All examinations	
Total number of examinations, N	36
Female:Male, N	27:9
Age (y)	11.2 (0.1–17–9)
Height (cm)	136.3 (50–182)
Weight (kg)	41.5 (3–126)
Contrast dose (ml)	2.5 (0.3–7.2)
Cirrhotic liver	
Total number of examinations, N	6
Female:Male, N	2:4
Age (y)	11.4 (4–11.4)
Height (cm)	141.7 (103–177)
Weight (kg)	36.7 (18–76)
Contrast dose (ml)	2.3 (0.8–4.8)
Non‐cirrhotic liver	
Total number of examinations, N	30
Female:Male, N	23:7
Age (y)	11.1 (0.1–17.9)
Height (cm)	135.0 (50–182)
Weight (kg)	42.5 (3–126)
Contrast dose (ml)	2.5 (0.3–7.2)

Descriptive data for all included examinations and for subgroups “cirrhotic” and “non‐cirrhotic” liver groups. Mean values presented with value range within parenthesis.

The age of the patients ranged from 0.1 to 17.9 years. Three (8%) were aged 0–2.0 years, 8 (22%) were 2.1–7.0 years, 5 (14%) were 7.1–12.0 years and 20 (56%) were 12.1–17.9 years.

No adverse reactions to the CEUS were observed.

As reference for the FLL diagnosis, histology results were available in 28% (10/36) of the cases. Follow‐up of at least two years with radiological imaging was available in 36% (13/36) of the cases. The radiological follow‐up was with CECT in 23% of the cases (3/13) and with CEMRI in 77% (10/13). Clinical follow‐up without radiologic imaging of at least two years was available in 14% (5/36). Two of these cases were followed up by a hepatologist for four and five years, respectively, until they were no longer considered at need for further follow‐up. The other three cases were followed up as part of regular appointments for other chronic medical issues. Follow‐up with radiologic imaging within two years was available in 22% (8/36) of the cases. Of these, two underwent a CECT after one and one and a half years, respectively, two had repeated CEUS examinations after three months and six months, one had a CEMRI after one year and three had non‐contrast US examinations performed within six months.

A total of 35 out of the 36 lesions (97%) were determined to be benign by this reference standard. One case was determined to be malignant.

Reference diagnosis in all cases, subdivided into a “cirrhotic” and “non‐cirrhotic” liver group, is presented in Table 2.

**Table 2:** Reference diagnosis

Reference diagnosis	Cirrhotic liver (*n* = 6)		Non‐Cirrhotic liver (*n* = 30)		Total (*n* = 36)	
	*n*	%	*n*	%	*n*	%
Benign						
Benign, uncharacterisable	2	33	11	37	13	36
FNH	1	17	10	34	11	30
Regenerative nodule	2	33	2	7	4	11
Cyst	0	0	4	13	4	11
Adenoma	1	17	0	0	1	3
Hematoma	0	0	1	3	1	3
Haemangiondothelioma	0	0	1	3	1	3
Malignant						
Klatskin tumour	0	0	1	3	1	3

FNH, Focal Nodular Hyperplasia.

Distribution of reference diagnosis for all examinations, subdivided into cirrhotic and non‐cirrhotic groups.

The overall agreement between the CEUS diagnosis and the reference diagnosis for benign vs malignant differentiation, where haemangioendothelioma was considered a benign diagnosis was 75% (27/36).

There was disagreement between CEUS and reference diagnosis in nine of the 36 cases (25%). In all of these cases, the investigations were determined to be of sufficient diagnostic quality, and the disagreement between CEUS and reference diagnosis did not seem to be attributed to any technical or visual problem with the examination. In one case, CEUS report suggested a malignant lesion but the reference showed it to be benign. In four cases, CEUS was inconclusive for the differentiation between benign and malignant lesions. In all of these cases, the reference diagnosis showed that the lesion was benign. In four cases, CEUS could not visualise any focal liver lesion. However, in three of these cases, the reference diagnosis showed that there was a benign lesion present and in one of the cases the reference showed that there was a malignant lesion present.

When analysing only the CEUS cases that were conclusive, leaving out the four inconclusive cases and the four cases where CEUS could not visualise the lesion, the overall agreement between CEUS and reference diagnosis for benign versus malignant differentiation was 96% (27/28). While sensitivity and positive predictive value could not be calculated, the specificity for correctly characterizing a lesion as benign was 96% and the negative predictive value was 100%.

When looking at specific diagnoses, agreement between CEUS diagnosis and reference diagnosis for the three most common diagnoses was as is shown in Table 3.

**Table 3:** Diagnosis agreement

Reference diagnosis	Agreement between CEUS and reference diagnosis (%)
FNH	91 (10/11)
Regenerative nodule	75 (3/4)
Cyst	100 (4/4)

FNH, Focal Nodular Hyperplasia.

Agreement between CEUS diagnosis and reference diagnosis for the three most common diagnoses. Presented as percentage with absolute numbers within parenthesis.

As previously explained, there was disagreement between the CEUS and the reference diagnosis in nine cases. Four of these were cases where CEUS could not characterise the lesion at hand and they were labelled as “inconclusive”. The remaining five cases, four of which were cases where CEUS could not visualise an existing lesion, and one case where CEUS incorrectly classified a benign lesion as malignant, are presented below in more detail. The case where a malignant lesion could not be visualised was re‐evaluated by the senior CEUS radiologist in our institution. The details of this case and the results from the re‐evaluation are presented separately below.

### Case of malignant lesion not visualised

1. Seventeen‐year‐old girl with no prior hepatic condition was referred to our institution from a local hospital where a medical investigation was initiated because of GI problems and elevated liver enzymes. In the local hospital, they performed several non‐contrast US, two CECTs and one CEMRI about three months prior to the CEUS at our institution. These examinations showed a previous portal thrombotisation and diffuse stricture‐inducing changes in the hilar region of the liver with secondary biliary duct strictures. One CEUS examination was also performed in the local hospital during this time and it could not visualise any FLL. A general liver biopsy (not from the hilar region) was without pathology.

Once in our institution, the examinations from the local hospital were reviewed and the changes in the hilar region were determined to be probably malignant. A PET‐CT was performed that showed hypermetabolic changes in the gallbladder – which was reported as probably inflammatory changes, but no other pathologic regions could be seen. Two weeks prior to the CEUS, a CEMRI was performed in our institution. This reported that the changes causing strictures were unclear and that no significant growth could be seen from the previous examinations about two months earlier – which together with apparent diffuse cystic changes in the right hepatic lobe and around the gallbladder could suggest a pathology other than cholangiocarcinoma. Tuberculosis or parasitic disease was suggested as differential diagnosis.

The primary objective of the CEUS performed in our institution was to aid the radiologist in performing a focal biopsy of the changes in the hilar region. Characterisation of the lesion was a secondary objective.

The CEUS reported that no FLL could be identified and no focal biopsy could be performed at that time. Two weeks after the CEUS, a laparoscopic biopsy was performed and showed a Klatskin tumour (hilar cholangiocarcinoma).

Secondary evaluation of the case showed that apart from non‐contrast US images and cineloops, only arterial phase cinenloops of the CEUS examination were stored. Hence, no complete evaluation of the CEUS examination could be made.

The secondary evaluation of the available images and cineloops, given the limited imaging, was in accordance with the original report – namely that no pathological enhancement pattern could be seen in the hilar region in the arterial phase.

### Other cases not visualised

1. Sixteen‐year‐old boy with prehepatic portal vein thrombosis and oesophageal varices, without cirrhosis. A routine follow‐up non‐contrast US examination, performed three weeks prior to the CEUS, reported an uncharacterisable FLL. A CEMRI one week prior to the CEUS reported the same. CEUS reported no visible FLL. Follow‐up with MRI after eight weeks showed no change and reported the lesion as a probable regenerative noduli. Follow‐up with CEMRI four years later reported the FLL as a probable regenerative noduli.
2. Fifteen‐year‐old girl with Budd–Chiari syndrome, without cirrhosis. A routine follow‐up with non‐contrast US examination reported a possible FLL. Three weeks later, a CEUS was performed and it reported the absence of any FLLs. CECT three months later reported an FLL consistent with the finding on non‐contrast US. It was reported as having a benign appearance but was not further characterisable. MRI two years later reported multiple lesions consistent with nodular regenerative hyperplasia associated with Budd–Chiari syndrome.
3. Four‐year‐old girl with tyrosinemia and cirrhosis with known nodular regenerative noduli. MRI performed abroad reported a new FLL and a CEUS was requested for further evaluation. No information on when the MRI was performed or if it was performed with contrast was available. CEUS reported no FLLs. An MRI abroad performed four and five years, respectively, after CEUS, reported a benign but otherwise non‐characterisable FLLs.

### Cases incorrectly classified as malignant

1. Seventeen‐year‐old boy referred to gastroenterologist due to obesity and elevated liver enzymes. Non‐contrast US shows lesion that could not be further characterised. CEUS was performed as the first imaging examination after US and the lesion was reported to be a possible hepatocellular carcinoma (HCC). MRI was performed seven weeks after the CEUS examination, and it reported the lesion as benign but non‐characterisable. No biopsy was performed. A follow‐up MRI after one year showed no change in appearance, and the lesion was still reported as benign and possibly an FNH or an atypical haemangioma. No more follow‐ups were done after this.

## Discussion

Our results indicate, in concordance with the available literature on adults,^5^, ^6^, ^12^, ^13^, ^14^ that CEUS is an accurate and relevant diagnostic tool for correctly identifying an FLL and for classifying it as benign also in children. The possible implication of this is that in select cases, further examinations and follow‐up can be reduced.

Of the few available paediatric studies, one of the largest^15^ reported a specificity and NPV for CEUS as a tool for correctly identifying an FLL as benign of 98% and 100%, respectively. Studies show that the diagnostic performance and accuracy is significantly increased for CEUS when comparing it to non‐enhanced US.^16^, ^17^ Our results are fairly in line with these, and also with the EFSUMB position statement on the paediatric use of CEUS.^5^ However, our calculations regarding specificity and NPV were analysed on a limited amount of data, which may reduce the generalisability of the study.

36% of the cases in our study were classified as “benign, uncharacterisable”, meaning that although the examination could not render a specific diagnosis it could provide the reassurance of reporting the lesion as benign. This is significant information for both the patient as well as the caregiver.

When looking at specific diagnoses amongst our data, CEUS seemed most reliable for the identification of FNHs, nodular hyperplasias and cysts. This is in accordance with a large prospective multicentre study,^14^ conducted on adults, where the accuracy for FNH diagnosis was as high as 98.3%.

The use of CEUS in these settings has the potential to reduce healthcare costs by avoiding further examinations (CT and/or MRI) beyond the CEUS examinations.^18^, ^19^ One Italian study^18^ found that in 87.6% of adult patients with FLLs, it would have been unnecessary to perform any further examinations following CEUS. Also, one should not underestimate the importance of being able to reduce patient and parent anxiety by hopefully providing the reassurance of a benign finding through a fast, non‐invasive and safe procedure.

CEUS has, in various studies on adults, shown to be comparable to MRI and CT in the identification and characterisation of FLLs.^20^, ^21^, ^22^, ^23^ The consensus so far is that the same applies to children.^5^, ^12^, ^24^

Although safe^19^, ^25^, ^26^ and reliable, CEUS has its limitations. In the guidelines and recommendations on hepatic use of CEUS from 2020 by EFSUMB in cooperation with the World Federation for Ultrasound in Medicine and Biology (WFUMB),^27^ there is comprehensive information on the subject. Here, one can find one very important restriction: CEUS should only be used to investigate single, or multiple but closely located focal lesions, since it is impossible to scan the whole liver at once in all the vascular phases. This means that it currently is not recommended in HCC surveillance in cirrhotic livers. Furthermore, as is the case with any US examination, the lesion of interest has to allow complete visualisation, which may not be the case where a FLL is located deep in the liver or obscured by bowel gas or the ribcage.

Our results highlight the possibilities of CEUS, but also show that there are challenges associated with its use. For instance, there were four cases where CEUS failed to identify an FLL, one of which turned out to be malignant. The secondary evaluation of this case concluded that there was not enough information stored to allow for a complete evaluation of the examination and that within the stored imaging, no FLL could be identified. This highlights two issues: one, CEUS examinations require standardised protocols for scanning and storage of these examinations to allow for secondary evaluation when needed, and two, the primary objective of the CEUS examination will vary from time to time and hence will affect the focus of the examination and the report. In our case where the Klatskin tumour that could not be visualised, the primary objective of the examination was not to characterise the lesion but rather to guide the radiologist in performing a biopsy of the pathologic area; this may have to do with the limitations of CEUS as explained above, but with the limitations inherent of this retrospective study this cannot be further clarified.

One curiosity regarding our data is that 75% of the examinations were performed on females. Theoretically, this could affect the generalisability of our study. We found no medical explanation for this skewness and consider it to be due to chance.

Also, there are cases where unusually high contrast doses were administered. In one case, 7.2 ml was given, which is three times the full dose of 2.4 ml. We found no explanation for this and can only assume that the radiologist in charge made the decision to repeat the doses in order to achieve a diagnostic examination.

Due to the retrospective nature of our study, one limitation is that there is information that is not available to us, which is a prospective study could be taken into account. For instance, all radiologists performing the CEUS examinations were experienced in CEUS but we do not have information regarding their specific experience in paediatrics. Considering that US in general and CEUS in particular is highly user dependent, inter‐user variability is a potential issue and subject for further prospective studies. Also, we have no specific information on the technical details regarding the ultrasound hardware and software, both of which could affect the quality of the examination and its interpretation. It is clear that histology is the ultimate reference standard. However, if the lesion has a low likelihood of malignancy, follow‐up by non‐invasive methods is preferred in our hospital. As we serve a tertiary referral centre for paediatric and adult hepatology, these patients stay under our surveillance as long as needed, that is until malignancy has been clearly ruled out in follow‐up.

There is the possibility that the interpretation of the CEUS examinations could be influenced by the diagnosis suggested by the previously performed CT or MRI introducing the possibility of bias.

The strengths of our study are that our source of information, the hospital electronic patient chart and the local PACS system are considered very reliable and no alternative sources of data exist for the information we required.

The radiologic department in our hospital was a very early adopter of CEUS for both adults and children alike. This ensured a working infrastructure around these examinations in the radiology department, where the examinations usually were performed, as well as in the clinical paediatric ward.

In summary, it could be argued that in certain cases CEUS in itself is sufficiently reliable for correct diagnosis, without the need for further imaging, thus avoiding radiation exposure and/or sedation as well, saving both time and cost. In other cases, it can be incorporated in the clinical workup alongside traditional imaging as CECT and CEMRI, providing additional diagnostic information.

## Conclusion

Our study reinforces that CEUS can be useful in the medical workup for the identification and classification of focal liver lesions in children.

## Authorship Statement

This is to acknowledge that all authors stated in the authorship listing conforms with the journal’s authorship policy, and that all authors are in agreement with the content of the submitted manuscript.

## Funding

No funding information is provided.

## Conflicts of Interest

Authors declare no conflicts of interest.

## Author Contributions

**Alvaro Torres:** Conceptualisation (equal); Data curation (lead); Formal analysis (equal); Investigation (equal); Methodology (equal); Project administration (lead); Writing‐original draft (lead); Writing‐review & editing (equal). **Seppo Koskinen:** Conceptualisation (equal); Data curation (equal); Formal analysis (equal); Investigation (equal); Methodology (equal); Project administration (equal); Supervision (lead); Visualisation (equal); Writing‐original draft (supporting); Writing‐review & editing (equal). **Henrik Gjertsen:** Conceptualisation (equal); Data curation (equal); Formal analysis (equal); Investigation (equal); Methodology (equal); Project administration (supporting); Supervision (equal); Validation (equal); Visualisation (equal); Writing‐original draft (supporting); Writing‐review & editing (equal). **Björn Fischler:** Conceptualisation (equal); Data curation (equal); Formal analysis (equal); Investigation (equal); Methodology (equal); Project administration (supporting); Supervision (equal); Validation (equal); Visualisation (equal); Writing‐original draft (supporting); Writing‐review & editing (equal).
